# High-throughput quantitative histology in systemic sclerosis skin disease using computer vision

**DOI:** 10.1186/s13075-020-2127-0

**Published:** 2020-03-14

**Authors:** Chase Correia, Seamus Mawe, Shane Lofgren, Roberta G. Marangoni, Jungwha Lee, Rana Saber, Kathleen Aren, Michelle Cheng, Shannon Teaw, Aileen Hoffmann, Isaac Goldberg, Shawn E. Cowper, Purvesh Khatri, Monique Hinchcliff, J. Matthew Mahoney

**Affiliations:** 1 Department of Internal Medicine, Division of Rheumatology, Northwestern University Feinberg School of Medicine, Chicago, IL USA; 2 Department of Neurological Sciences, University of Vermont Larner College of Medicine, HSRF 408 149 Beaumont Avenue, Burlington, VT 05405 USA; 3 Department of Experimental Radiation Oncology, The University of Texas MD Anderson Cancer Center, Houston, TX 77030 USA; 4 Institute for Public Health and Medicine, Chicago, IL USA; 5 Department of Preventive Medicine, University of Vermont Larner College of Medicine, Burlington, VT USA; 6 Yale School of Medicine, Department of Medicine, Section of Rheumatology, Allergy & Immunology, New Haven, CT USA; 7 Department of Dermatology, Yale University School of Medicine, New Haven, CT USA; 8 Department of Pathology, Yale University School of Medicine, New Haven, CT USA; 9 Department of Medicine (Biomedical Informatics - Research Institute for Immunity, Transplantation and Infection) and of Biomedical Data Science, Stanford University, Palo Alto, CA USA; 10 Department of Computer Science, University of Vermont, Burlington, VT USA

**Keywords:** Computer vision, Systemic sclerosis, Scleroderma, Histology, Modified Rodnan skin score, Outcomes, Outcome measures, Deep neural network, AlexNet, Quantitative image features

## Abstract

**Background:**

Skin fibrosis is the clinical hallmark of systemic sclerosis (SSc), where collagen deposition and remodeling of the dermis occur over time. The most widely used outcome measure in SSc clinical trials is the modified Rodnan skin score (mRSS), which is a semi-quantitative assessment of skin stiffness at seventeen body sites. However, the mRSS is confounded by obesity, edema, and high inter-rater variability. In order to develop a new histopathological outcome measure for SSc, we applied a computer vision technology called a deep neural network (DNN) to stained sections of SSc skin. We tested the hypotheses that DNN analysis could reliably assess mRSS and discriminate SSc from normal skin.

**Methods:**

We analyzed biopsies from two independent (primary and secondary) cohorts. One investigator performed mRSS assessments and forearm biopsies, and trichrome-stained biopsy sections were photomicrographed. We used the AlexNet DNN to generate a numerical signature of 4096 quantitative image features (QIFs) for 100 randomly selected dermal image patches/biopsy. In the primary cohort, we used principal components analysis (PCA) to summarize the QIFs into a *Biopsy Score* for comparison with mRSS. In the secondary cohort, using QIF signatures as the input, we fit a logistic regression model to discriminate between SSc vs. control biopsy, and a linear regression model to estimate mRSS, yielding *Diagnostic Scores* and *Fibrosis Scores,* respectively. We determined the correlation between Fibrosis Scores and the published Scleroderma Skin Severity Score (4S) and between Fibrosis Scores and longitudinal changes in mRSS on a per patient basis.

**Results:**

In the primary cohort (*n* = 6, 26 SSc biopsies), Biopsy Scores significantly correlated with mRSS (R = 0.55, *p* = 0.01). In the secondary cohort (*n* = 60 SSc and 16 controls, 164 biopsies; divided into 70% training and 30% test sets), the Diagnostic Score was significantly associated with SSc-status (misclassification rate = 1.9% [training], 6.6% [test]), and the Fibrosis Score significantly correlated with mRSS (R = 0.70 [training], 0.55 [test]). The DNN-derived Fibrosis Score significantly correlated with 4S (R = 0.69, *p* = 3 × 10^− 17^).

**Conclusions:**

DNN analysis of SSc biopsies is an unbiased, quantitative, and reproducible outcome that is associated with validated SSc outcomes.

## Background

Skin fibrosis is the systemic sclerosis (SSc) clinical hallmark. The modified Rodnan skin score (mRSS) is a validated, semi-quantitative measure of skin fibrosis [1]. Although the most commonly used primary outcome in SSc clinical studies, the mRSS is limited by the need for specialized training to decrease intra- and inter-rater variability [2], the coarseness of the measure, and confounding by obesity and edema. Much research has focused on direct or indirect methods for SSc dermatopathology quantification, including plicometry [3, 4], durometry [5], and high frequency ultrasound [6]. Histopathological assessment of fibrosis using dermal thickness measurements [7] and quantification of α-smooth muscle actin [8] have also been explored. Finally, molecular surrogates of SSc skin disease, such as gene expression and serum proteomic signatures, have also been proposed [9, 10]. For example, a serum proteome signature [10] and the Scleroderma Skin Severity Score (4S) [11] are both associated with mRSS. The 4S is a skin gene expression signature that was identified and validated using publicly available heterogeneous transcriptome data from seven independent cohorts of patients with SSc from six clinical centers. We demonstrated that 4S is positively correlated with mRSS in each of the seven cohorts and that 4S change in the first year is associated with mRSS change in the second year [11]. However, all of these approaches are limited by the need to measure ‘omics’ data, which are expensive and not readily available, or are labor-intensive assays requiring human-expert review. To date, none of these approaches have supplanted the mRSS. The development and validation of a quantitative, reproducible, and scalable method to predict SSc disease severity is a large, unmet need.

Computer vision holds the promise to radically augment our current pathology practices using scalable and objective algorithms to assess tissue properties that currently require a highly trained human expert. A recent advance in computer vision is the development of deep neural networks (DNNs), which are algorithms for image quantification and classification. DNNs have revolutionized computer vision, achieving human-level performance at image recognition while reducing analytic times and standardizing interpretation. In dermatological conditions including psoriasis and skin cancer, DNNs have recently shown expert-level pattern recognition from macroscopic and microscopic images of affected skin [12–14]. However, one major barrier to developing DNNs is the large number, typically millions, of training examples required to train the network, which itself has millions of parameters [15]. For problems with many fewer training examples, including rare diseases like SSc, training a DNN de novo is not feasible [16]. To overcome this limitation, the machine learning community has developed a procedure called *transfer learning* whereby researchers use a pre-trained DNN algorithm as a pre-processing pipeline before applying a machine learning strategy that robustly classifies variables on smaller sample sizes. In this study, we used the publicly available AlexNet DNN to pre-process SSc skin biopsy images before applying multivariate statistical analyses to establish that histological features from stained dermal biopsy sections from patients with SSc and healthy control participants can precisely quantify SSc dermal histological variation.

**Fig. 1:**
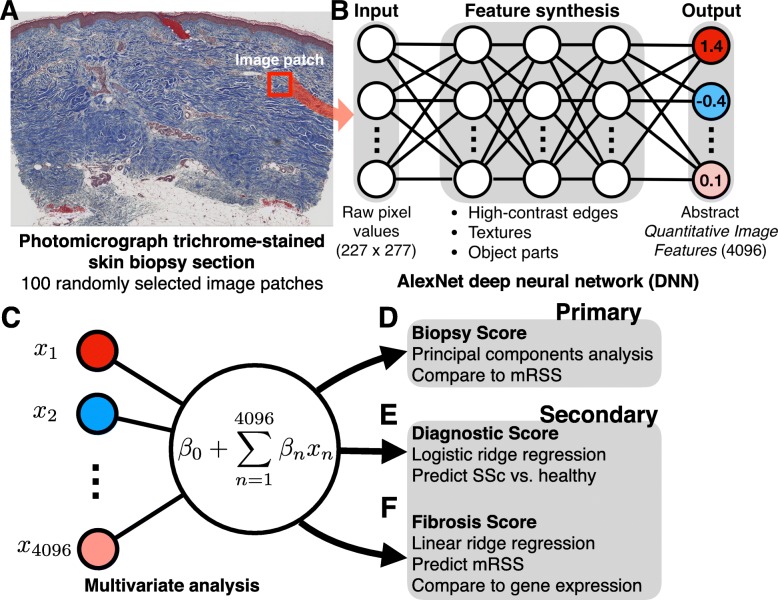
Deep neural network (DNN) processing of trichrome-stained skin sections. A) Trichrome-stained skin biopsy sections from patients with SSc and healthy controls were photomicrographed at 40x resolution. To sample variability in tissue structure, we randomly selected 100 *image patches* from the dermis (red box) corresponding to ~ 0.16 mm^2^. B) Each image patch was used as input to the AlexNet DNN. AlexNet maps the raw pixel values of the input image to a series of more complex image features. The final output is a 4096-dimensional signature of abstract Quantitative Image Features that were used for subsequent multivariate statistical analyses. C) Principal components analysis and multivariate analyses using QIF as the predictor variables were conducted in order to develop, D) a Biopsy Score, E) a Diagnostic Score, F) a Fibrosis Score that was compared to mRSS and skin gene expression biomarkers

A DNN image analysis produces a high-dimensional numerical signature of *Quantitative Image Features* (QIFs) that quantify image properties, from simple properties, such as high contrast edges between stained regions, to complex properties, such as extended textural patterns (Fig. 1). These QIFs, while abstract mathematical properties of an image, are a powerful tool for analyzing natural and biomedical images [12, 13, 15]. For example, DNNs perform on par with dermatopathologist review of stained skin lesion sections for diagnosis of nodular basal cell carcinomas, dermal nevi, and seborrheic keratoses [14]. In this study, we applied DNN algorithms to stained sections of skin biopsies from patients with SSc and from healthy participants [17, 18]. Our goal was to test the hypothesis that DNN algorithms are sensitive to histological variation in skin biopsies and to assess the association with markers of SSc disease severity including the mRSS and two SSc gene expression biomarkers: the SSc four-gene biomarker [9] and the 4S gene expression biomarker [11]. The results herein demonstrate that computer vision applied to stained SSc biopsy sections may be a novel, quantitative, robust, and scalable SSc outcome.

## Patients and methods

### Participant recruitment and inclusion criteria

Research partners provided written informed consent in accordance with the Declaration of Helsinki Protocols and Northwestern University Institutional Review Board guidelines (STU00004428). Patients fulfilled the 2013 American College of Rheumatology SSc criteria [19]. Medical histories, physical exams, and biopsies were completed at clinical visits. SSc disease duration was defined as the interval between the first non-Raynaud symptom attributed to SSc and the time of the baseline skin biopsy. Early SSc was defined as < 24-month SSc disease duration. Serum anti-topoisomerase I, anti-centromere, and anti-RNA polymerase III antibody titers were measured by indirect immunofluorescence at Specialty Laboratories, Valencia, CA. Healthy control participants were recruited from the Northwestern University clinical and research communities to match the age (within 10 years), race and sex of an SSc patient as previously described [11, 17, 18].

### Clinical assessment, dermal punch biopsies and histopathological analysis

One rheumatologist performed mRSS assessments and paired 4 mm dermal, punch biopsies of the non-dominant dorsal forearm. Biopsies were repeated at 6-, 12-, 24-, and 36-months proximal to preceding biopsies. One biopsy was fixed and embedded in paraffin, and a 4-μm section was stained with Masson’s trichrome and photomicrographed using a Leica SCN400 slide scanner (Wetzlar, Germany) at 40x magnification. Masson’s Trichrome was used because it is a standard connective tissue stain that highlights collagen structure. The other paired biopsy was placed in RNAlater and used for skin gene expression analyses as previously described [17].

### Deep neural network feature extraction

Leica™ SCN format images of stained sections were converted into PNG files and transformed into QIFs using AlexNet in the Matlab Neural Network Toolbox [15] (Fig. 1a, b). DNNs are a sequence of mathematical transformations organized in *layers*; the outputs of each layer are the inputs of the subsequent layer (Fig. 1b and Supplementary Methods). The lowest layers capture primitive image properties, such as intensity and color contrasts, while progressively higher levels capture more complex properties such as patterns and textures. Because AlexNet was originally trained to classify “natural” images (e.g., animals and humanmade objects), the highest layers are specialized to those classes. However, the intermediate layers contain QIFs that are not highly specialized, and therefore can be used for other computer vision tasks in a process called *transfer learning* [16]. QIFs were extracted from the intermediate ‘fully connected layer 6’ from AlexNet, which outputs 4096 QIFs. We recently showed that these 4096 features work well for transfer learning in histopathological studies of kidney disease [20]. AlexNet has a fixed input size of 227 × 227 pixels (~ 0.16 mm^2^), thus randomly sampled 227 × 227-pixel image patches from the dermis of each section were transformed into QIFs. Each QIF in the dataset was normalized to have a mean of zero and a standard deviation of one.

### Principal component analysis for dimension reduction in primary cohort

Because of the high dimensionality of QIF signatures, principal components analysis (PCA) was used to reduce the 4096 QIFs into a single summary score, termed *Image Patch Score*, in arbitrary units (AU) (Table 1) [21]. The Image Patch Score is a weighted combination of all 4096 QIFs that captures the most variation across the 2600 image patches [21]. Image patches with more distant scores are more dislike each other across all QIFs, and therefore more dislike each other histologically. One-way analysis of variance was used to assess the variance of *Image Patch Scores* using Matlab. In order to generate one summary score for each biopsy, we calculated the mean of the 100 Image Patch Scores, termed the *Biopsy Score* (AU) (Table 1). We assessed the Spearman correlation, corrected for repeated measures, between the biopsy score and the mRSS using the R package “rmcorr”.

**Table 1:** Description of Analyses and Terms

Term	Analysis tool	Purpose
Image Patch Score	Principal Component Analysis was applied to the 4096 Quantitative Image Features (QIF) generated by the deep neural network (DNN) algorithm for each of the 100 image patches/biopsy	To quantitatively summarize the variance in SSc biopsy histology
Biopsy Score	The mean of the 100 Image Patch Scores for each biopsy section	Used as a discovery tool to assess the utility of applying DNN algorithms to stained SSc biopsies. Defining the Biopsy score as the mean of the 100 Image Patch Scores enabled generation of one quantitative histologic score for each biopsy section
Diagnostic Score	Logistic regression	To identify QIF that are associated with SSc versus health control biopsy
Fibrosis Score	Linear regression	To identify QIF that are associated with mRSS

### Logistic regression model to classify SSc vs. normal biopsies

In order to determine whether DNN analyses can “see” SSc skin disease, we built and validated an SSc diagnostic model. Using QIFs as predictor variables, we trained a logistic regression model to distinguish between SSc and healthy skin. We divided a secondary cohort into training (70% patients) and test (30% patients) sets and stratified by disease status and high (> 20) and low (≤20) mRSS to ensure that: 1) neither set had a disproportionate number of low mRSS cases that could be difficult to distinguish from normal, and 2) the regression model for mRSS (next section) had both high and low mRSS cases for training. In order to eliminate within-subject bias, all biopsies from one patient went into either the training or test set. Because of the large QIF number, we fit the logistic regression model using ridge regression, which is a standard method for preventing model over-fitting by penalizing overly complex models [22]. Ridge regression required setting a *hyperparameter*, lambda, that mediates the strength of the penalty (i.e. how strongly the penalty trades off between fitting the training data and keeping the model coefficients small). We set lambda through ten-fold cross-validation (10-FCV) on the training data set. Briefly, 10-FCV splits the training data into ten equal parts and fits the model ten times, each time holding out one part for testing. The generalization performance of the model is measured by its ability to predict the correct class labels for the held-out 10% of the data. To eliminate within-subject and within-class bias, the 10-FCV parts were stratified by subject and disease status. Because there were many more SSc subjects than healthy controls, we weighted the data points so that each class had equal weight, which is standard for unequal group sizes [23]. We selected lambda from a grid of 16 values logarithmically spaced between 10^− 5^ (weak penalty) and 10^3^ (strong penalty). For every choice of lambda and every image patch, the logistic regression model generates a linear score that is mapped to a probability that the image patch comes from a patient with SSc as follows:

$$ Y={\beta}_0+\sum \limits_{i=1}^{4096}{\beta}_i{X}_i $$

$$ Prob\;\left(\mathrm{SSc}\right)=\frac{1}{1+{e}^{-Y}}, $$

where *X*_*i*_ is the i^th^ QIF, *β*_*i*_ is the regression coefficient, and Y is mapped to probability by the *logistic function*.

In order to integrate from image patches to the full biopsy, we averaged the linear scores over all image patches within a biopsy before mapping with the logistic function to obtain a lambda-dependent *Diagnostic Score* (Table 1). We selected lambda based on the value whose Diagnostic Scores generalized the best at discriminating between SSc patients and healthy controls most strongly by a two-sample t-statistic. Any sample with Diagnostic Score > 0.5, i.e. more probably SSc than control, was classified as SSc by the model. All Diagnostic Scores were calculated using a cross-validation model that did not contain that sample (i.e. using *out-of-bag* data). To test the diagnostic model, the coefficients of the logistic regression models for the optimal lambda were averaged over all ten folds of the training data (model averaging) and used to generate Diagnostic Scores for the 30% held-out test set. Misclassification rates were defined as the number of incorrect predictions divided by the total number of predictions. Receiver operating characteristic (ROC) curves were computed to determine the quality of the binary classification model. Briefly, ROC curves compute the true positive rate (TPR) and false positive rate (FPR) as a function of changing the classification threshold from Diagnostic Score = 1, where the model is 100% confident the biopsy is from a patient with SSc, to Diagnostic Score = 0, where the model is 0% confident the biopsy is not SSc (i.e., 100% confident the biopsy is from a healthy individual). The area under the ROC curve (AUC) is a measure of how well separated the groups are, where AUC = 1 means that *at some Diagnostic Score threshold* the classes are perfectly separated, while AUC = 0.5 means that the model is no better than random guessing. *Note that it is possible to have AUC = 1, but also non-zero misclassification rate at a fixed threshold; For example, it is possible that the classes are separated perfectly at a Diagnostic Score of 0.6, but not at a fixed* a priori *Diagnostic Score of 0.5.* All model fitting and ROC analysis were performed using custom scripts and the Matlab functions ‘fitclinear’ and ‘perfcurve’.

### Linear regression model and association with mRSS and local skin score

To build and validate a regression model to predict fibrosis, we proceeded identically as above, except that we used a linear vs. logistic regression model. Briefly, we used the same training, test, and cross-validation partitions, data weights, and lambda values as above with ridge-penalized linear regression to predict mRSS using image patch-level QIFs. For each image patch and value of lambda, the linear model predicts a value for mRSS as a linear combination of the QIF levels plus an *intercept* that captures the mean mRSS of the study cohort. To integrate from image patches to biopsies, we averaged the predicted scores for every image patch within a biopsy to obtain a final *Fibrosis Score* (Table 1). Because of the averaging and the ridge penalty, the predicted range mRSS was compressed relative to the measured values, which is well known to occur with ridge regression. However, because we ultimately seek an independent score for fibrosis, the total scale is arbitrary. Thus, we selected lambda as the value within the training set that achieved the highest Pearson correlation between the predicted Fibrosis Scores and the true mRSS. All Fibrosis Scores were calculated using a cross-validation model that did not contain that sample (i.e. using out-of-bag data). To test the fibrosis model on completely independent data, the coefficients of the linear regression models for the optimal lambda were averaged over all ten folds of the training data and used to generate Fibrosis Scores for the 30% held out test set. The Pearson correlation between the test Fibrosis Scores and mRSS was calculated to assess the generalization performance of the model. Because the mRSS scores were not normally distributed, we also computed the Spearman correlations between Fibrosis Scores and mRSS as a secondary measure of association. We also tested the correlation between Fibrosis Scores and the local skin score at the site of the biopsy. To avoid circularity, we computed this correlation using the 30% held out test data set, which was not used to train the Fibrosis score. All model fitting was performed in Matlab using custom scripts and the Matlab function ‘fitrlinear’.

### Association with gene expression correlates of skin disease severity

We compared Fibrosis Score to two gene expression signatures that have been shown to correlate well with the mRSS in previous studies: the Scleroderma Skin Severity Score (4S) [11] and the SSc four gene biomarker [9]. To compute 4S for a skin biopsy, we mapped microarray probe intensities to genes. Sample probe intensities were quantile normalized. Genes with multiple probe mappings were then assigned the intensity of the mean of their respective probe intensities. The mean of underexpressed 4S-signature genes was subtracted from the mean of overexpressed 4S-signature genes to generate 4S for each biopsy [11]. The final set of raw scores for each biopsy were scaled to a mean of zero and standard deviation of one. The correlation between 4S and the Fibrosis Score for each biopsy was determined. As described, all Fibrosis Scores were either the out-of-bag scores from cross-validation (training biopsies) or the score from the final averaged model (test biopsies).

To compare Fibrosis Scores to the four SSc biomarker genes [9], we extracted all microarray probes that matched the genes *COMP*, *THBS1*, *IFI44*, and *SIGLEC1*. Four probes for *COMP*, *THBS1*, and *IFI44* were included in our expression data set. We then computed the Pearson correlation between Fibrosis Scores and the expression of these probes.

### Longitudinal analyses of skin biopsies

In order to explore whether the Fibrosis Score was sensitive to change within a patient, we fit a linear mixed effects (LME) model predicting mRSS as a function of Fibrosis Score. This analysis accounted for between-subject differences in average Fibrosis Score (modeled as a random intercept), so a significant association of Fibrosis score to mRSS in this model indicates that within-subject variation in mRSS is correlated with within-subject changes in Fibrosis Score. To avoid circularity, we fit this model only on the 30% held-out testing data, which were not used to develop the Fibrosis Score. Model fitting was performed in Matlab using the function ‘fitlme’.

## Results

We applied DNN analyses to two cohorts. The primary cohort consisted of 26 biopsies from six SSc patients while the secondary cohort consisted of 148 biopsies from 60 patients and 16 biopsies from 16 healthy participants (Table 2). Patients, the majority with early dcSSc, and healthy controls tended to be Caucasian, middle-aged women.

**Table 2:** Clinical characteristics of patients with systemic sclerosis

Clinical Characteristic	Primary Cohort	Secondary Cohort	
Mean ± Standard Deviation or as indicated	SSc patients (n = 6)	SSc patients (***n*** = 60)	Healthy controls (***n*** = 16)
Age	54 ± 6	50 ± 11	42 ± 12
Women (n, %)	5 (83%)	52 (87%)	13 (81%)
Race, Caucasian (n, %)	5 (83%)	45 (75%)	12 (75%)
SSc disease duration (months)*	11 ± 3	38 ± 51	
dcSSc (n, %)	6 (100%)	42 (70%)	
mRSS (median, IQR)	15 (8)	15 (8)	
Serum autoantibodies (n, %)			
Anticentromere	0	4 (7%)	
Anti-RNA polymerase III	3 (50%)	15 (25%)	
Anti-topoisomerase I (Scl-70)	2 (33%)	19 (31%)	

*Months between date of onset of first non-Raynaud systemic sclerosis (SSc) symptom and date of skin biopsy. *dcSSc* Diffuse cutaneous SSc

### Exploratory analysis shows association between DNN-derived QIFs and mRSS

**Fig. 2:**
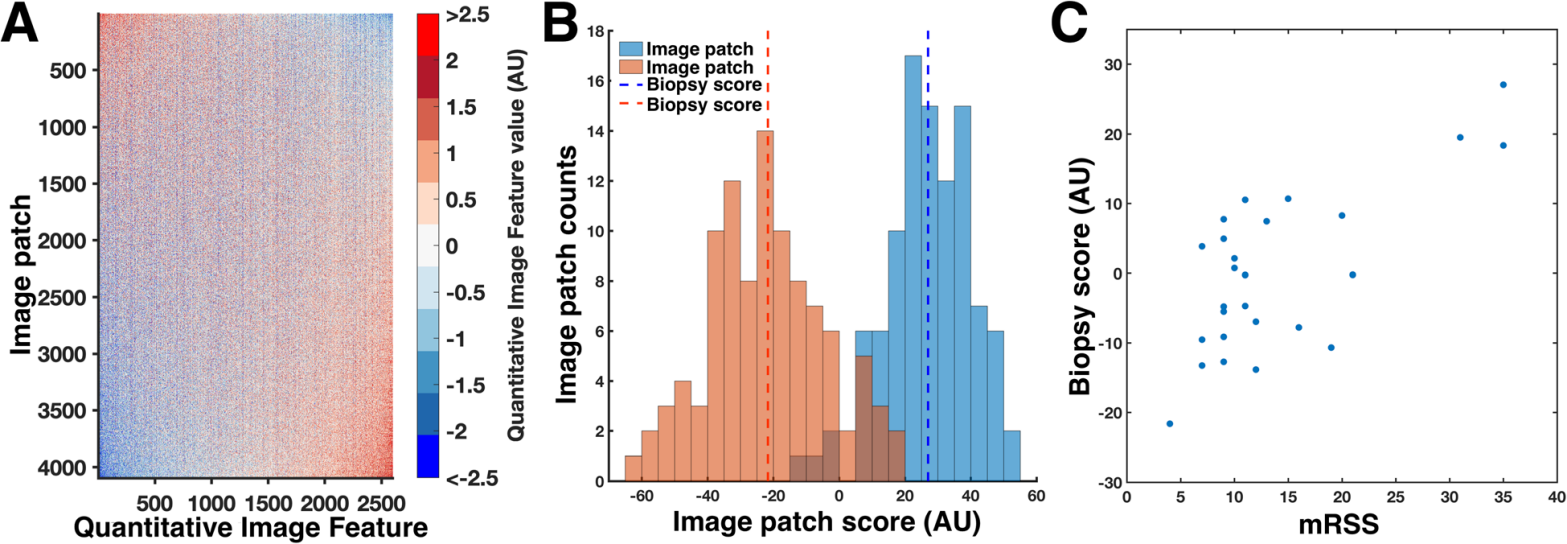
Association between DNN-derived signatures and mRSS. A) To visualize the distribution of Quantitative Image Features (QIFs) for the primary cohort, a heat map was generated with columns consisting of image patches (26 patients × 100 image patches) and rows consisting of 4096 QIFs. For display, the rows and columns of the heat map were sorted by the first principal component, which highlights both the correlation among features across the set of image patches and the differences in QIF signatures across image patches. B) The distribution of the 100 *Image Patch Scores* for two biopsies is shown for a patient with low (orange), and high (blue) modified Rodnan Skin Score (mRSS). The mean *Image Patch Score* for each biopsy, termed the *Biopsy Score* (dotted vertical line) is shown. C) *Biopsy Scores* significantly correlated with mRSS (R = 0.55, p = 0.010, repeated measures test)

In an exploratory study, we assessed the association between QIFs and SSc skin fibrosis using PCA to reduce data complexity. To visualize the extremes of the fibrosis distribution, we plotted histograms of the Image Patch Scores from the patients with the highest and lowest mRSS values. We observed segregation indicating that fibrosis differences can be detected as differences in the Image Patch Score distributions (Fig. 2b). We performed one-way ANOVA across all 2600 image patches grouped by biopsy, and biopsy accounted for 30% of the total variance in the Image Patch Scores (*p* < 10^− 100^, one-way ANOVA). Thus, while there was significant variation in Image Patch Scores within a biopsy, there were significant differences between biopsies in the mean value of their biopsy scores. Next, we assessed the correlation between the DNN-derived Biopsy Score (mean of the 100 Image Patch Scores per biopsy) and mRSS (Fig. 2c, Spearman’s rho =0.55, *p* = 0.010, repeated measures test). This result demonstrated that DNN-derived QIF can “see” clinical differences in skin disease extent from a single punch biopsy.

### DNN features contain diagnostic and skin fibrosis severity information

To extend these results, we studied an independent secondary cohort of biopsies from 60 patients and 16 healthy participants (164 biopsies total). Because data-driven Biopsy Scores significantly correlated with mRSS in the primary cohort, we hypothesized that, in a sufficiently powered data set, it would be possible to distinguish SSc from healthy control biopsies and to explicitly predict mRSS. Because PCA is an exploratory technique rather than a predictive modeling technique, we adopted a regression-based approach in the secondary cohort.

**Fig. 3:**
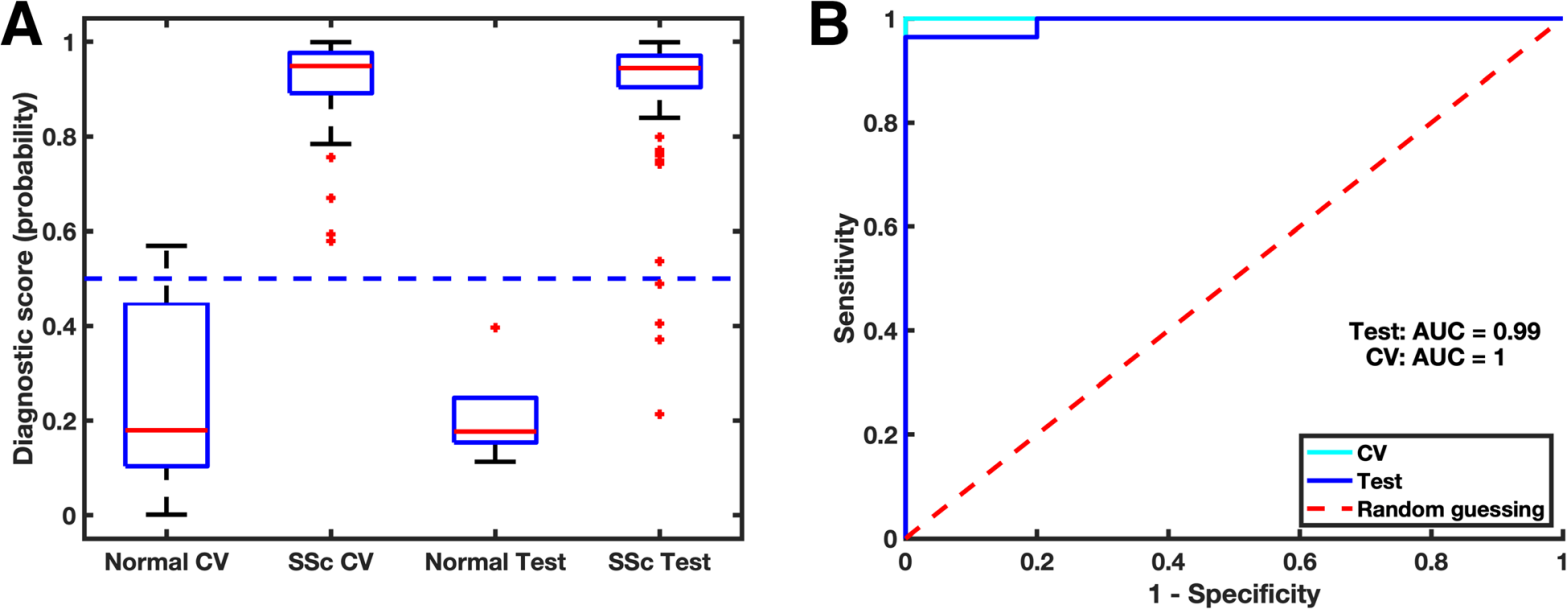
Prediction of SSc status in a secondary cohort. A) We developed and independently tested a logistic regression model composed of Quantitative Image Features (QIFs) to predict SSc vs. healthy control. The model output was a Diagnostic Score, i.e. the predicted probability that the image was from a patient with SSc. Box plots of the of the cross-validation (CV) and independently tested Diagnostic Scores show significant separation between groups. Using a hard threshold of 0.5, the model achieved a 1.9% misclassification rate using cross validation in the training data and 6.6% on the independent testing data set. B) As an alternative visualization of the model performance, the ROC curves for the logistic regression model show that the model achieves high area under the curve (AUC) for both CV (AUC =1.00) and testing (AUC =0.99)

To generate and test regression models, we divided the secondary cohort into a training set (103 biopsies, 70% of subjects) and a test set (61 biopsies, 30% of subjects). The training and test sets were stratified by subject (i.e. all biopsies from a single patient were either in the training or test set). To test whether the DNN could distinguish patients from controls, we fit a logistic regression model using the 4096 QIFs from the training set as the independent variables and the Diagnostic Score (SSc vs. healthy) as the outcome variable. Using 10-fold cross-validation within the training set, the model had a misclassification rate of 1.9% at a Diagnostic Score of 0.5. The model was then validated in the test set achieving a misclassification rate of 6.6% at a Diagnostic Score = 0.5. In both the training and testing sets, the healthy biopsies had low Diagnostic Scores compared to SSc biopsies demonstrating the face validity of our model (Fig. 3a). Moreover, ROC curves for the training and test sets show that the Diagnostic Scores robustly discriminates between patient and control biopsies (AUC = 1.00 [training], 0.99 [testing]; Fig. 3b). Note that the perfect AUC but non-zero misclassification rates in the training set is due to the fact that all Diagnostic Scores for normal skin are below the lowest SSc score, but some Diagnostic Scores for normal skin are higher than the fixed 0.5 cutoff (see also Patients and Methods). These data establish proof-of-principle that SSc skin can be discriminated from normal skin using DNN QIFs.

**Fig. 4:**
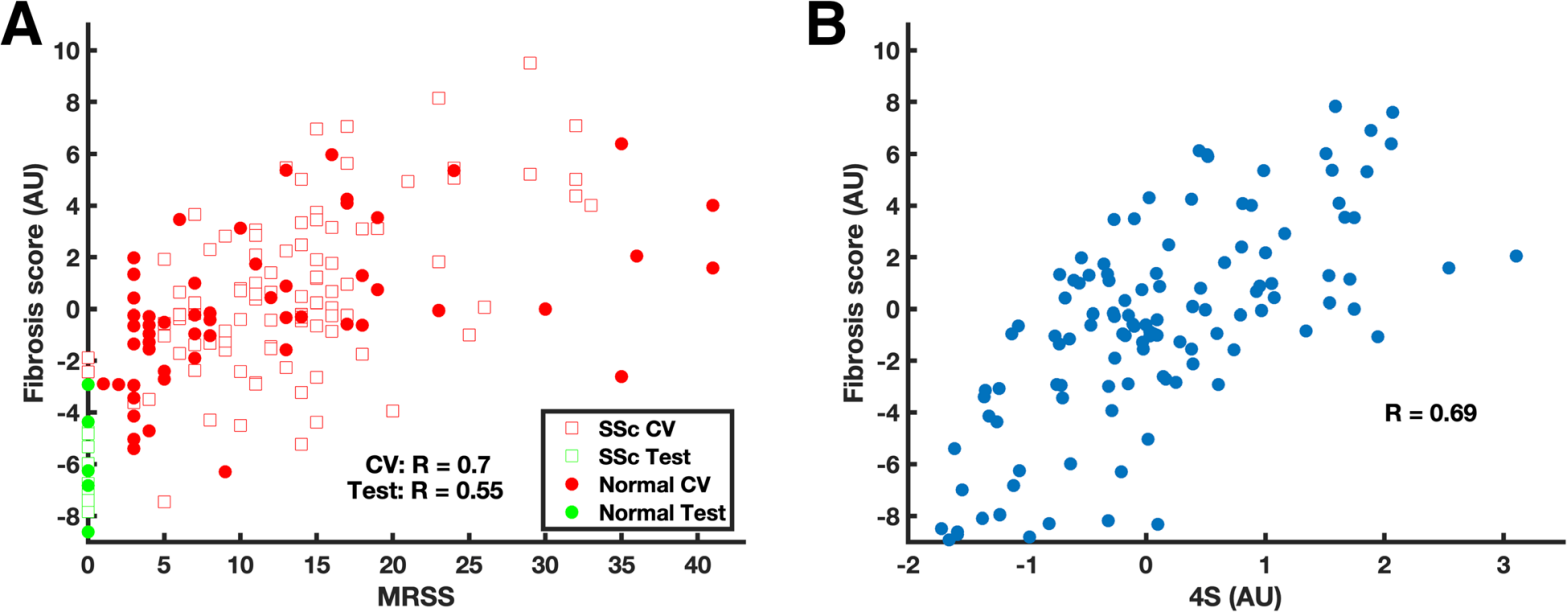
Prediction of fibrosis and association with molecular pathology. A) We developed and independently tested a linear regression model composed of Quantitative Image Features (QIFs) to predict mRSS. The model output was a Fibrosis Score measured arbitrary units (AU). Using cross validation (CV) on the training data set, the Fibrosis Score strongly correlated with modified Rodnan Skin Score (mRSS) (R = 0.70, open squares). This correlation generalized to the independent test set (R = 0.55, *p* = 5.3 × 10^− 6^; solid dots). B) The DNN-derived Fibrosis Score significantly correlated with the validated Scleroderma Skin Severity Score (4S) (R = 0.69, *p* = 2.9 × 10^− 17^)

Next, using the training set, we fit a linear regression model using the 4096 QIFs as independent variables to predict mRSS (see Methods). Using 10-fold cross-validation within the training set, we found that the Fibrosis Score strongly correlated to mRSS (R = 0.70; Spearman’s rho = 0.65), and this generalized to the test set (R = 0.55, *p* = 5.3 × 10^− 6^; Spearman’s rho = 0.64, *p* = 2.9 × 10^− 8^; Fig. 4a). The comparable correlations with mRSS between training and testing sets indicate that the fibrosis model is also well calibrated and not overfitted. The Fibrosis Score also significantly correlated with the local skin score at the site of the biopsy in the test data set (R = 0.56, *p* = 6.1 × 10^− 6^). These data establish proof-of-principle that the extent of skin fibrosis can be assessed using DNN QIFs.

To investigate the utility of the DNN-derived Fibrosis Score for SSc skin disease quantification, we correlated the Fibrosis Score with 4S, a validated skin gene expression signature of skin disease severity. For 115 biopsies, we had calculated a 4S score from a previous study [11]. The Fibrosis Score was strongly correlated with 4S (R = 0.69, p = 2.9 × 10^− 17^; Fig. 4b), demonstrating that histological information potentially overlaps underlying molecular pathological information. The Fibrosis Score was also significantly correlated with the expression of *COMP* (R = 0.39,0.22; *p* = 9.2 × 10^− 5^), *THBS1* (R = 0.61; *p* = 2.5 × 10^− 11^), and *IFI44* (R = 0.28; *p* = 0.005), three genes reported as biomarkers of skin disease severity (Supp. Fig. 1).

Finally, to investigate whether changes in Fibrosis Score correlated with changes in mRSS with a patient, we fit a linear mixed effects model with subject as a random effect to account for between-subject variability in mRSS (Supp. Table 1). Fibrosis Score was significantly associated with mRSS in this model (*p* = 0.0005), demonstrating that changes in Fibrosis Score correlated with changes in mRSS within subject.

## Discussion

The lack of a robust, reproducible and quantitative method to accurately assess skin disease severity in patients with SSc plagues clinical studies. An ideal outcome should be sensitive to the underlying pathogenic mechanisms, scalable, and robust, i.e. free of inter-rater variability and confounding. Herein, we applied established DNN computer vision algorithms to a single, stained dermal biopsy section from patients with SSc and healthy participants to test the hypothesis that DNN analysis can precisely quantify SSc dermal histological variance. In an exploratory analysis, we generated QIFs that we used to develop two SSc skin disease metrics, termed the Image Patch Score (i.e. the first PC for the DNN-derived 4096 QIFs for each image patch), and the Biopsy Score (i.e. the mean of the 100 Image Patch Scores per biopsy). We observed strong correlation between the Biopsy Score and mRSS suggesting that our approach had validity and that extension studies were warranted. Next, in a large independent cohort of patient and control biopsies, we tested the ability of DNN-derived scores to identify (Diagnostic Score) and quantify (Fibrosis Score) SSc skin disease, and we correlated Fibrosis Score with 4S, a validated SSc skin gene expression biomarker, in order to further test the validity of our DNN approach. Together, our results demonstrate that DNN-derived SSc skin disease metrics are a feasible, quantitative, reproducible and scalable SSc outcome.

One of the key advantages of the computer vision framework used in this study is the reproducibility and scalability of automated analysis. In the community setting, mRSS is not routinely measured. In the research setting, the mRSS continues to be an imprecise measure with high inter-rater variability even among rheumatologists [2, 24, 25]. Similar limitations render manual histopathological scoring of SSc dermal fibrosis suboptimal [5]. With robust scoring using DNNs, the problem of assessing SSc skin disease can be moved to a fully automated pipeline that will generate exactly reproducible results for every image. Moreover, because the pipeline is purely computational, the analysis is scalable. This latter point could allow for much wider catchment for SSc clinical trials. Using a well-validated computer vision system for scoring SSc skin disease as a primary outcome measure would allow many more medical centers to participate in clinical trials. Investigators could perform a simple skin punch biopsy and fix biopsies in formalin. In particular, it would eliminate the need for specially trained mRSS scorers at the point-of-care. Instead, paraffin-embedded, formalin-fixed tissue could be pooled from multiple centers for staining and assessment at a single center. In the near term, such a strategy could be used as a secondary outcome measure in order to validate the DNN-based approach in clinical trials.

The conclusions that can be drawn from our study are limited because our study population is enriched for patients with early dcSSc and therefore does not represent the full spectrum of SSc (lcSSc vs. dcSSc, early vs. late disease) or healthy control (differing sex, age and race/ethnicity) skin. Thus, our diagnostic and fibrosis prediction models must be interpreted as proof-of-concept that DNNs can extract clinically relevant information from histological images. The development and deployment of a classification or histological scoring model in a clinical setting will require external independent validation data sets to account for differences in patient demographics and skin biopsy collection, processing, and staining methods that exist at different sites, as these could influence DNN-derived QIFs. While we were unable to evaluate the effects of these potential confounders in the present study, this single-site study provides evidence that these technical issues can be surmounted with sufficient pooling of data from multiple sites, for example as an adjunct analysis for large clinical trials. A second limitation is that, although our study cohort includes longitudinal biopsies from the same patient, and we stratified by subject in the training and testing sets to avoid within-subject bias, we were not powered to fit a longitudinal model. In fact, we only averaged 3.3 biopsies per SSc research participant at the time of this analysis due to staggered recruitment, patient deaths, loss to follow-up and patient refusal for additional biopsies. However, we do find that our Fibrosis Score, which was trained in a cross-sectional model, was associated with mRSS after accounting for between-subject variability in mean mRSS. This result provides confidence that a DNN-derived biopsy scores can be clinically meaningful, although more studies are requires to systematically optimize the prediction model. To this end, it is important to recognize the sources of variability between our Fibrosis Scores and mRSS, which, in principle, include biological variation in the Fibrosis Score that is not captured by mRSS, technical artifacts in the Fibrosis Score that are not biological, within-subject variation in tissue structure as a function of biopsy site, and the inherent variability of the mRSS. Addressing these sources of variation will require a detailed understanding of the histological features that underlie QIF signatures. Future studies should predict tissue-level endpoints, such as the number of myofibroblasts, dermal thickness, biopsy weight, or infiltrating immune cell numbers in addition to mRSS, in order to establish a mechanistic understanding of DNN-dervied QIFs. While beyond the scope of this proof-of-concept study, work is ongoing to address these questions.

Despite the above limitations, we stress that our results show that the pattern of collagen structure in a *single punch biopsy* can be used to reliably predict the extent of fibrosis over the whole body. This suggests that there is subtle variation at a single site that is predictive of fibrosis at distant sites. The DNN captures this variation in an unbiased QIF signature. Thus, our results demonstrate that DNN readouts are a useful high-throughput data source, akin to ‘omics’ data, for SSc histopathology. It is increasingly appreciated that there is a need to integrate data from SSc patients, from molecular measures to clinical outcomes, in order to more fully capture and quantify SSc disease heterogeneity [26, 27]. With DNN analysis of stained biopsy sections, quantitative histology can be readily incorporated into system biological analysis of SSc, where it can be integrated with ‘omic’ readouts, physiological measures, and laboratory tests [26, 27]. The results of this study are an important step towards the development of new SSc skin disease outcome measures and provide a scalable path to integrating histology into the systems biological study of SSc.

## Conclusions

Unlike the mRSS, computer vision applied to stained skin biopsy sections from patients with SSc is an unbiased, quantitative and reproducible SSc outcome for skin disease assessment.

## Supplementary information

**Additional file 1.** Supplemental methods AR&T 2019.

**Additional file 2 Figure S1.** Correlation between the Fibrosis Score and the SSc Four-Gene Biomarker. The correlation between the Deep Neural Network-derived Fibrosis Score and three of four genes (that passed filter criteria) from the SSc Four Gene Biomarker are displayed.

**Additional file 3 Table S1.** Local arm skin scores at the time of biopsy performance Secondary Cohort.

## Data Availability

Data sharing is not applicable to this article as no datasets were generated or analyzed during the current study.

## Abbreviations

4S: Scleroderma Skin Severity Score
AU: Arbitrary units
AUC: Area under the curve
dcSSc: diffuse cutaneous systemic sclerosis
DNN: Deep neural network
FCV: Fold cross-validation
mRSS: modified Rodnan Skin Score
PCA: Principal components analysis
QIF: Quantitative image feature
ROC: Receiver operating characteristic
SSc: systemic sclerosis
