# Supplementary material for: High-throughput quantitative histology in systemic sclerosis skin disease using computer vision
Source: Arthritis Res Ther. 2020 Mar 14;22:48. doi: 10.1186/s13075-020-2127-0 (PMC7071594; doi:10.1186/s13075-020-2127-0)
Supplement: Supplementary file 1 — Additional file 1. Supplemental methods AR&T 2019. [file 13075_2020_2127_MOESM1_ESM.docx]

Patients and Methods:

SSc disease duration was defined as the interval between the first non-Raynaud symptom attributed to SSc and the time of the baseline skin biopsy. Early SSc was defined as <24-month SSc disease duration. Serum anti-topoisomerase I, anti-centromere, and anti-RNA polymerase III antibody titers were measured by indirect immunofluorescence at Specialty Laboratories, Valencia, CA. Healthy control participants were recruited from the Northwestern University clinical and research communities to match the age (within 10 years), race and sex of an SSc patient as previously described {Lofgren, 2016 #640;Hinchcliff, 2013 #416;Hinchcliff, 2018 #426}.

*Deep neural network feature extraction*

Lowest layers capture primitive image properties (e.g. intensity and color contrasts), higher layers capture more complex properties (e.g. patterns and textures) with the highest layer used for image classification per design (e.g., animal or objects). However, intermediate layers consist of QIFs that can be used in other computer vision tasks via *transfer learning* {Goodfellow, 2016 #344}. The AlexNet intermediate ‘fully connected layer 6’ which outputs 4096 QIFs has recently been shown to work well for transfer learning in other DNN histological studies {Sheehan, 2019 #1406}. Logistic regression:

Briefly, 10-FCV splits the training data into ten equal parts and fits the model ten times, each time holding out one part for testing. The generalization performance of the model is measured by its ability to predict the correct class labels for the held-out 10% of the data. To eliminate within-subject and within-class bias, the 10-FCV parts were stratified by subject and disease status. Because there were many more SSc subjects than healthy controls, we weighted the data points so that each class had equal weight, which is standard for unequal group sizes {Strutz, 2016 #1360}. We selected lambda from a grid of 16 values logarithmically spaced between 10^-5^ (weak penalty) and 10^3^ (strong penalty). For every choice of lambda and every image patch, the logistic regression model generates a linear score that is mapped to a probability that the image patch comes from a patient with SSc as follows:


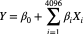


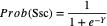
,

where
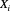
 is the i^th^ QIF,
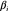
 is the regression coefficient, and Y is mapped to probability by the *logistic function*.

*Linear model to predict fibrosis in validation cohort*

Because of the averaging and the ridge penalty, the predicted range mRSS was compressed relative to the measured values, which is well known to occur with ridge regression. However, because we ultimately seek an independent score for fibrosis, the total scale is arbitrary. Thus, we selected lambda as the value within the training set that achieved the highest Spearman correlation between the predicted Fibrosis Scores and the true mRSS. All Fibrosis Scores were calculated using a cross-validation model that did not contain that sample (i.e. using out-of-bag data). To test the fibrosis model on independent data, the coefficients of the linear regression models for the optimal lambda were averaged over all ten folds of the training data and used to generate Fibrosis Scores for the 30% held out test set.

*Logistic regression to classify SSc vs. normal biopsies*

All model fitting and ROC analysis were performed in Matlab using custom scripts and the Matlab functions ‘fitclinear’ and ‘perfcurve’.

*Linear regression model and association with mRSS*

All model fitting was performed in Matlab using custom scripts and the Matlab function ‘fitrlinear’.
