# Supplementary figures and images for: High-throughput quantitative histology in systemic sclerosis skin disease using computer vision
Source: Arthritis Res Ther. 2020 Mar 14;22:48. doi: 10.1186/s13075-020-2127-0 (PMC7071594; doi:10.1186/s13075-020-2127-0)

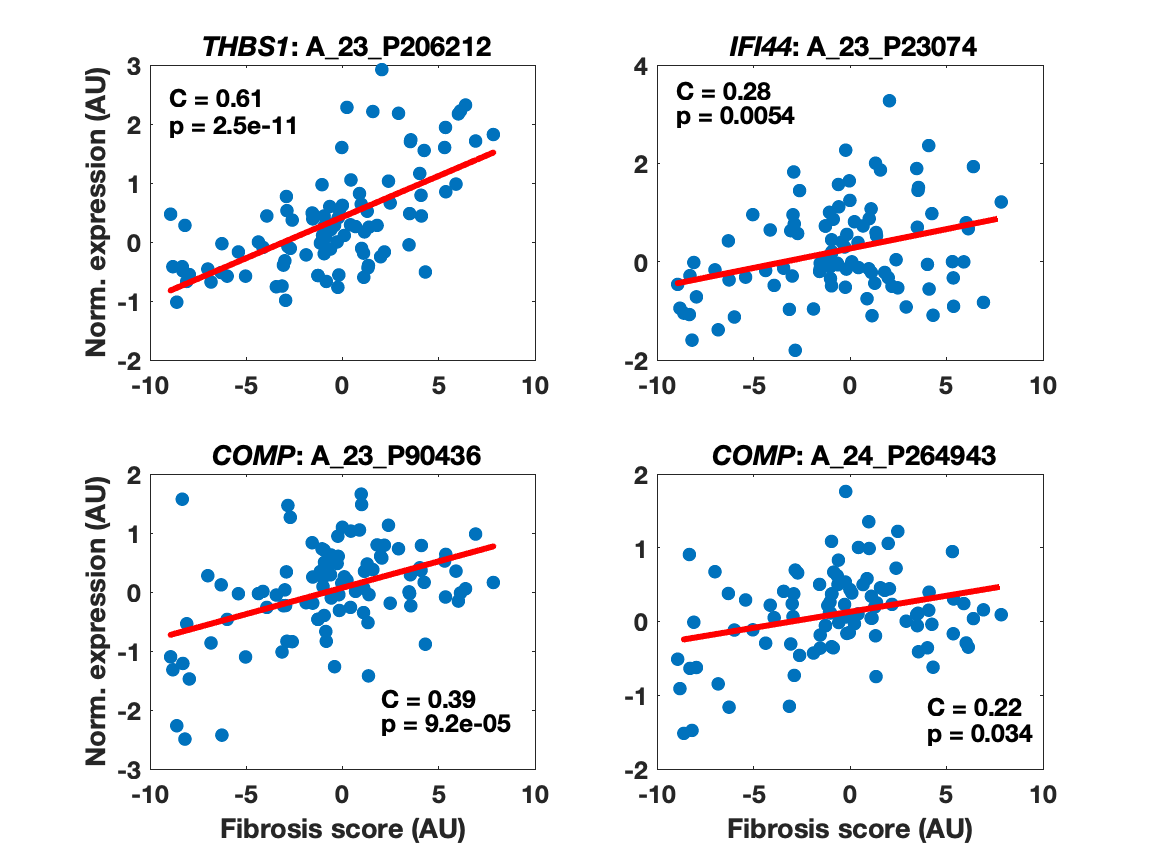

Supplement: Supplementary file 2 — Additional file 2 Figure S1. Correlation between the Fibrosis Score and the SSc Four-Gene Biomarker. The correlation between the Deep Neural Network-derived Fibrosis Score and three of four genes (that passed filter criteria) from the SSc Four Gene Biomarker are displayed. [file 13075_2020_2127_MOESM2_ESM.tif]
