# Supplementary material for: High-throughput quantitative histology in systemic sclerosis skin disease using computer vision
Source: Arthritis Res Ther. 2020 Mar 14;22:48. doi: 10.1186/s13075-020-2127-0 (PMC7071594; doi:10.1186/s13075-020-2127-0)
Supplement: Supplementary file 3 — Additional file 3 Table S1. Local arm skin scores at the time of biopsy performance Secondary Cohort. [file 13075_2020_2127_MOESM3_ESM.docx]

| **Supplementary Table 1: Local arm skin scores at the time of biopsy performance Secondary Cohort** | | |
| --- | --- | --- |
| Research Participant | Biopsy time point | Local arm skin score |
| 1 | baseline | 2 |
| 1 | 6 months | 2 |
| 1 | 2 months | 1 |
| 1 | 24 months | 2 |
| 1 | 36 months | 1 |
| 2 | baseline | 2 |
| 4 | baseline | 0 |
| 4 | 6 months | 0 |
| 4 | 2 months | 0 |
| 4 | 24 months | 0 |
| 5 | baseline | 2 |
| 5 | 6 months | 1 |
| 7 | baseline | 0 |
| 7 | 6 months | 0 |
| 7 | 2 months | 0 |
| 7 | 24 months | 0 |
| 7 | 36 months | 0 |
| 12 | baseline | 1 |
| 12 | 6 months | 1 |
| 12 | 2 months | 1 |
| 12 | 24 months | 1 |
| 12 | 36 months | 1 |
| 13 | baseline | 1 |
| 14 | baseline | 1 |
| 14 | 6 months | 1 |
| 14 | 2 months | 1 |
| 14 | 24 months | 0 |
| 14 | 36 months | 0 |
| 15 | baseline | 3 |
| 15 | 6 months | 3 |
| 15 | 2 months | 2 |
| 16 | baseline | 1 |
| 16 | 6 months | 1 |
| 16 | 2 months | 1 |
| 16 | 24 months | 1 |
| 16 | 36 months | 0 |
| 18 | baseline | 1 |
| 18 | 36 months | 1 |
| 19 | baseline | 1 |
| 19 | 6 months | 1 |
| 19 | 2 months | 1 |
| 19 | 24 months | 1 |
| 19 | 36 months | 1 |
| 20 | baseline | 1 |
| 20 | 6 months | 1 |
| 21 | baseline | 1 |
| 21 | 6 months | 1 |
| 21 | 2 months | 0 |
| 21 | 24 months | 0 |
| 21 | 36 months | 0 |
| 23 | baseline | 0 |
| 23 | 6 months | 0 |
| 23 | 2 months | 0 |
| 23 | 24 months | 0 |
| 23 | 36 months | 0 |
| 25 | baseline | 1 |
| 25 | 6 months | 1 |
| 25 | 2 months | 0 |
| 25 | 24 months | 0 |
| 25 | 36 months | 0 |
| 26 | baseline | 1 |
| 26 | 6 months | 1 |
| 26 | 2 months | 1 |
| 26 | 24 months | 1 |
| 26 | 36 months | 0 |
| 27 | baseline | 2 |
| 27 | 6 months | 1 |
| 27 | 2 months | 2 |
| 27 | 24 months | 1 |
| 27 | 36 months | 0 |
| 29 | baseline | 3 |
| 30 | baseline | 2 |
| 30 | 6 months | 1 |
| 30 | 2 months | 1 |
| 30 | 24 months | 0 |
| 30 | 36 months | 0 |
| 31 | baseline | 1 |
| 32 | baseline | 0 |
| 32 | 6 months | 0 |
| 32 | 2 months | 0 |
| 32 | 24 months | 0 |
| 33 | baseline | 1 |
| 33 | 6 months | 1 |
| 33 | 2 months | 0 |
| 33 | 24 months | 0 |
| 34 | 24 months | 0 |
| 34 | 36 months | 0 |
| 35 | baseline | 0 |
| 35 | 6 months | 0 |
| 35 | 24 months | 0 |
| 36 | baseline | 0 |
| 36 | 6 months | 0 |
| 37 | baseline | 1 |
| 37 | 24 months | 0 |
| 38 | baseline | 1 |
| 39 | baseline | 2 |
| 40 | baseline | 2 |
| 40 | 2 months | 1 |
| 41 | baseline | 0 |
| 41 | 6 months | 0 |
| 42 | baseline | 1 |
| 42 | 6 months | 0 |
| 42 | 2 months | 0 |
| 42 | 24 months | 1 |
| 42 | 36 months | 0 |
| 43 | baseline | 2 |
| 44 | baseline | 1 |
| 44 | 6 months | 1 |
| 44 | 2 months | 1 |
| 44 | 24 months | 0 |
| 45 | baseline | 1 |
| 45 | 6 months | 1 |
| 45 | 2 months | 1 |
| 45 | 24 months | 0 |
| 45 | 36 months | 1 |
| 47 | baseline | 1 |
| 47 | 6 months | 1 |
| 47 | 2 months | 1 |
| 48 | baseline | 1 |
| 48 | 2 months | 1 |
| 48 | 24 months | 0 |
| 48 | 36 months | 0 |
| 49 | baseline | 1 |
| 49 | 24 months | 1 |
| 50 | baseline | 1 |
| 50 | 6 months | 1 |
| 50 | 2 months | 1 |
| 50 | 24 months | 0 |
| 50 | 36 months | 0 |
| 51 | baseline | 1 |
| 52 | baseline | 1 |
| 52 | 6 months | 1 |
| 52 | 2 months | 1 |
| 52 | 24 months | 0 |
| 52 | 36 months | 0 |
| 53 | baseline | 0 |
| 53 | 6 months | 0 |
| 54 | baseline | 1 |
| 54 | 2 months | 1 |
| 54 | 36 months | 0 |
| 55 | baseline | 1 |
| 55 | 6 months | 2 |
| 55 | 2 months | 1 |
| 55 | 24 months | 1 |
| 55 | 36 months | 1 |
| 56 | baseline | 1 |
| 56 | 6 months | 1 |
| 56 | 2 months | 0 |
| 56 | 36 months | 0 |
| 58 | baseline | 0 |
| 58 | 2 months | 0 |
| 58 | 24 months | 0 |
| 58 | 36 months | 0 |
| 59 | baseline | 0 |
| 59 | 6 months | 0 |
| 59 | 36 months | 0 |
| 60 | baseline | 0 |
| 61 | baseline | 1 |
| 61 | 2 months | 0 |
| 61 | 24 months | 0 |
| 61 | 36 months | 0 |
| 62 | baseline | 0 |
| 63 | baseline | 1 |
| 64 | baseline | 1 |
| 65 | baseline | 2 |
| 66 | baseline | 1 |
| 66 | 6 months | 2 |
| 66 | 2 months | 2 |
| 66 | 24 months | 1 |
| 66 | 36 months | 0 |
| 67 | baseline | 1 |
| 67 | 6 months | 1 |
| 68 | baseline | 0 |
| 68 | 6 months | 1 |
| 68 | 2 months | 1 |
| 68 | 24 months | 0 |
| 68 | 36 months | 0 |
| 69 | baseline | 0 |
| 69 | 6 months | 0 |
| 69 | 2 months | 0 |
| 69 | 24 months | 0 |
| 69 | 36 months | 0 |
| 71 | baseline | 0 |
| 71 | 6 months | 1 |
| 71 | 2 months | 1 |
| 71 | 36 months | 0 |
| 73 | baseline | 1 |
| 73 | 24 months | 0 |
| 77 | baseline | 3 |
| 77 | 6 months | 3 |
| 84 | baseline | 0 |
| 84 | 6 months | 0 |
| 84 | 2 months | 0 |
| 84 | 24 months | 0 |
